# Supplementary material for: Brazilian germplasm of winter squash (Cucurbita moschata D.) displays vast genetic variability, allowing identification of promising genotypes for agro-morphological traits
Source: PLoS One. 2020 Jun 9;15(6):e0230546. doi: 10.1371/journal.pone.0230546 (PMC7282630; doi:10.1371/journal.pone.0230546)
Supplement: S1 Table — (DOCX) [file pone.0230546.s001.docx]

| **Supplementary table** **1**. Multi-categorical descriptors used in the assessment of the C. moschata germplasm maintained by BGH-UFV | |
| --- | --- |
| **Phase/organ** | **Descriptors** |
| **Vegetative** | Growth habit, stem colour (SC), intensity of leaf green (ILG), leaf silvering (LS), intensity of leaf silvering (ILS), leaf serration (LS), presence of trichomes in the leaves (PAL), amount of trichomes in the adaxial surface of leaves (ATT), amount of trichomes in the abaxial surface of leaves (ATL), leaf recess (LR), presence of trichomes in the petiole (PTP), amount of trichomes in the petiole (ATP), and green intensity of male pedicel (GIMP). |
| **Fruit** | Format of fruits (FF), format of peduncle (FP), number of colours of fruit peel (NPC), topography of fruit surface (FT), format of floral scar (FFS), peel texture (PT), predominant colour of fruit peel (PC), and depth of fruits slices (DFS). |
| **Seed** | Seed format (SF), aspect of seed tegument (AST), seed tegument texture (STT), colour of seed tegument (CST), and colour of seed border (CSB). |
