# Supplementary material for: Brazilian germplasm of winter squash (Cucurbita moschata D.) displays vast genetic variability, allowing identification of promising genotypes for agro-morphological traits
Source: PLoS One. 2020 Jun 9;15(6):e0230546. doi: 10.1371/journal.pone.0230546 (PMC7282630; doi:10.1371/journal.pone.0230546)
Supplement: S2 Table — (DOCX) [file pone.0230546.s002.docx]

| **Supplementary table** **2**. Result of principal component analysis showing the fifteen principal components and the relative contribution of traits in each component | | | | | | | | | | | | | | | |
| --- | --- | --- | --- | --- | --- | --- | --- | --- | --- | --- | --- | --- | --- | --- | --- |
| Traits | PC 1 | PC 2 | PC 3 | PC 4 | PC 5 | PC 6 | PC 7 | PC 8 | PC 9 | PC 10 | PC 11 | PC 12 | PC 13 | PC 14 | PC 15 |
| S21 | 1.10 | 0.10 | 0.03 | 0.08 | 0.17 | 0.03 | 1.52 | 0.25 | 0.02 | 7.48 | 1.03 | 2.04 | 2.19 | 0.01 | 0.59 |
| S28 | 0.07 | 0.24 | 0.01 | 1.30 | 0.12 | 0.01 | 3.22 | 1.36 | 0.19 | 2.83 | 0.20 | 0.88 | 1.69 | 3.02 | 6.53 |
| LMV14 | 0.00 | 0.01 | 0.35 | 2.26 | 0.21 | 3.99 | 4.82 | 0.00 | 0.06 | 2.12 | 4.21 | 0.00 | 1.84 | 0.04 | 0.56 |
| LMV21 | 0.66 | 0.02 | 0.09 | 2.98 | 0.15 | 3.21 | 2.11 | 0.82 | 0.18 | 1.22 | 4.83 | 0.28 | 1.89 | 1.10 | 0.48 |
| DDF | 0.21 | 1.30 | 0.94 | 0.30 | 0.13 | 0.47 | 4.29 | 0.01 | 0.00 | 0.06 | 2.40 | 0.01 | 1.15 | 0.14 | 5.39 |
| NFP | 1.66 | 2.88 | 2.44 | 0.90 | 3.02 | 0.43 | 1.33 | 3.77 | 0.18 | 0.08 | 0.85 | 0.00 | 3.20 | 0.05 | 0.41 |
| MF | 11.76 | 0.32 | 0.17 | 0.39 | 0.03 | 0.01 | 0.20 | 0.06 | 0.04 | 0.91 | 0.00 | 0.09 | 0.03 | 0.28 | 0.54 |
| PF | 4.38 | 0.25 | 2.70 | 0.07 | 0.85 | 0.81 | 1.42 | 5.81 | 0.00 | 0.90 | 0.82 | 0.03 | 1.97 | 0.23 | 0.25 |
| HF | 6.10 | 1.46 | 1.64 | 1.08 | 2.96 | 0.11 | 0.18 | 1.26 | 1.32 | 1.32 | 1.20 | 0.13 | 0.00 | 0.07 | 0.03 |
| DF | 8.84 | 0.14 | 0.48 | 0.09 | 0.45 | 0.00 | 0.20 | 0.05 | 0.32 | 0.82 | 1.73 | 0.40 | 0.35 | 0.21 | 0.50 |
| TFP | 3.27 | 0.04 | 0.25 | 0.21 | 0.00 | 0.82 | 0.05 | 0.74 | 0.25 | 1.07 | 0.00 | 7.42 | 1.00 | 2.11 | 0.01 |
| RFP | 0.26 | 0.07 | 3.39 | 3.21 | 0.78 | 0.08 | 0.00 | 0.13 | 0.83 | 0.50 | 1.19 | 0.22 | 0.05 | 0.04 | 0.07 |
| RP | 0.18 | 1.00 | 8.51 | 1.07 | 1.49 | 0.01 | 0.13 | 0.56 | 1.76 | 0.05 | 0.26 | 0.20 | 0.00 | 0.03 | 0.70 |
| PT | 1.93 | 1.58 | 0.34 | 2.20 | 1.12 | 0.05 | 0.50 | 0.68 | 0.70 | 3.37 | 3.67 | 0.39 | 0.59 | 0.04 | 0.07 |
| DIC | 8.81 | 0.27 | 0.37 | 0.00 | 1.21 | 0.01 | 0.25 | 2.06 | 1.11 | 0.00 | 0.02 | 0.29 | 0.08 | 0.45 | 0.06 |
| TC | 0.86 | 0.93 | 0.11 | 0.19 | 0.17 | 0.02 | 0.13 | 4.45 | 0.00 | 0.60 | 3.50 | 0.33 | 0.51 | 0.40 | 0.38 |
| L | 0.36 | 0.26 | 0.53 | 0.01 | 0.62 | 0.00 | 0.13 | 0.91 | 1.15 | 0.32 | 0.55 | 0.03 | 0.79 | 4.83 | 7.31 |
| NSF | 2.41 | 4.48 | 3.53 | 0.47 | 0.00 | 0.09 | 0.01 | 0.17 | 0.04 | 0.33 | 0.00 | 0.11 | 3.55 | 3.61 | 0.00 |
| MSF | 4.99 | 4.40 | 1.49 | 0.05 | 0.33 | 0.63 | 0.62 | 1.19 | 0.47 | 0.60 | 0.13 | 0.05 | 2.60 | 0.15 | 0.18 |
| MS/F | 2.22 | 5.25 | 3.21 | 0.14 | 0.50 | 0.33 | 0.03 | 0.67 | 0.15 | 0.36 | 0.47 | 0.36 | 0.11 | 0.00 | 1.20 |
| MOHS | 4.65 | 0.01 | 0.25 | 0.00 | 1.13 | 0.30 | 2.79 | 0.20 | 3.50 | 0.87 | 1.31 | 0.49 | 0.16 | 4.01 | 0.28 |
| PS | 0.00 | 6.15 | 5.79 | 1.44 | 4.07 | 0.00 | 0.27 | 0.37 | 0.57 | 0.09 | 0.76 | 0.07 | 0.24 | 0.00 | 0.12 |
| SL | 2.08 | 0.69 | 3.80 | 0.55 | 0.88 | 0.01 | 0.03 | 3.24 | 3.82 | 2.00 | 0.72 | 0.91 | 0.34 | 0.38 | 0.01 |
| SW | 5.20 | 0.07 | 0.97 | 0.02 | 0.24 | 0.37 | 1.01 | 0.09 | 3.96 | 2.95 | 0.41 | 0.41 | 0.79 | 0.15 | 0.17 |
| ST | 1.98 | 1.00 | 0.01 | 0.07 | 1.39 | 0.06 | 0.84 | 1.48 | 0.49 | 0.01 | 0.71 | 0.47 | 0.02 | 7.03 | 0.26 |
| SOC | 0.10 | 0.13 | 5.93 | 0.26 | 1.38 | 0.29 | 1.71 | 0.39 | 0.07 | 1.65 | 0.01 | 1.70 | 0.00 | 0.01 | 1.14 |
| SOP | 0.00 | 6.13 | 5.57 | 1.30 | 3.90 | 0.06 | 0.20 | 0.25 | 0.52 | 0.18 | 1.31 | 0.08 | 0.29 | 0.00 | 0.28 |
| GH | 1.46 | 1.85 | 0.04 | 0.20 | 0.00 | 1.85 | 1.33 | 0.06 | 0.61 | 0.50 | 3.96 | 0.57 | 0.08 | 0.47 | 0.10 |
| SC | 0.01 | 0.52 | 2.23 | 0.55 | 3.98 | 4.94 | 2.20 | 1.40 | 0.02 | 3.27 | 5.17 | 3.01 | 16.29 | 1.03 | 0.22 |
| ILG | 0.36 | 1.19 | 0.37 | 4.40 | 1.03 | 6.84 | 1.24 | 2.38 | 5.28 | 2.47 | 4.82 | 1.76 | 1.11 | 2.68 | 2.83 |
| LSQ1 | 0.10 | 0.07 | 2.03 | 0.07 | 0.07 | 1.81 | 0.06 | 2.11 | 0.97 | 0.00 | 4.52 | 0.00 | 0.53 | 0.00 | 4.59 |
| ILS | 0.50 | 0.89 | 1.00 | 0.04 | 7.02 | 1.20 | 0.13 | 0.17 | 0.82 | 0.32 | 3.65 | 0.54 | 0.43 | 0.06 | 0.10 |
| LSQ2 | 1.21 | 0.91 | 0.60 | 0.14 | 1.12 | 5.57 | 1.68 | 0.25 | 2.49 | 0.61 | 0.31 | 0.40 | 1.80 | 11.88 | 0.60 |
| PTL | 0.06 | 0.32 | 0.77 | 0.21 | 0.07 | 1.30 | 3.01 | 0.99 | 0.02 | 5.98 | 2.43 | 0.01 | 0.74 | 0.55 | 3.52 |
| ATT | 0.38 | 10.91 | 2.47 | 17.20 | 5.62 | 0.81 | 0.93 | 0.66 | 0.18 | 0.40 | 0.17 | 0.52 | 0.16 | 1.62 | 0.06 |
| ATL | 0.02 | 7.27 | 2.78 | 13.03 | 0.85 | 1.99 | 0.12 | 0.62 | 0.87 | 0.21 | 1.34 | 1.06 | 0.38 | 1.04 | 2.86 |
| LR | 0.91 | 10.30 | 6.96 | 9.00 | 8.98 | 1.10 | 6.11 | 3.71 | 2.71 | 3.01 | 2.33 | 2.82 | 1.54 | 3.17 | 2.11 |
| PTP | 0.61 | 0.21 | 1.09 | 0.08 | 0.00 | 0.02 | 1.04 | 1.99 | 2.10 | 6.15 | 0.01 | 7.99 | 0.18 | 0.52 | 0.08 |
| ATP | 0.15 | 9.45 | 2.37 | 17.16 | 5.11 | 0.67 | 1.81 | 1.40 | 0.39 | 0.28 | 0.27 | 0.46 | 0.24 | 1.73 | 0.16 |
| GIMP | 0.63 | 2.31 | 0.27 | 0.61 | 0.22 | 0.94 | 0.29 | 4.61 | 7.92 | 1.02 | 4.27 | 6.92 | 0.17 | 0.91 | 1.42 |
| FF | 2.01 | 2.42 | 8.11 | 4.47 | 8.54 | 4.60 | 4.99 | 2.95 | 7.15 | 8.89 | 10.03 | 9.58 | 2.58 | 7.61 | 4.31 |
| FP | 0.28 | 0.68 | 0.93 | 1.13 | 0.53 | 4.96 | 0.80 | 3.40 | 5.13 | 8.92 | 3.05 | 3.21 | 0.69 | 2.09 | 2.75 |
| NPC | 0.91 | 0.06 | 0.01 | 1.03 | 0.20 | 13.76 | 0.03 | 2.85 | 0.91 | 0.48 | 0.57 | 0.16 | 0.02 | 0.17 | 2.45 |
| FT | 2.19 | 0.74 | 1.57 | 1.46 | 2.66 | 4.22 | 10.07 | 1.27 | 9.31 | 0.00 | 0.24 | 2.22 | 1.39 | 0.98 | 5.83 |
| FFS | 2.46 | 2.70 | 0.35 | 1.10 | 7.61 | 4.41 | 1.48 | 3.76 | 9.12 | 5.45 | 3.87 | 9.62 | 9.75 | 6.96 | 4.33 |
| PTQ | 1.00 | 0.73 | 5.40 | 0.44 | 3.60 | 4.62 | 3.56 | 1.32 | 4.31 | 4.46 | 6.01 | 1.89 | 18.54 | 4.00 | 7.47 |
| PC | 1.28 | 1.55 | 1.51 | 2.57 | 5.81 | 14.23 | 6.85 | 5.05 | 3.45 | 6.42 | 2.55 | 12.53 | 2.77 | 7.45 | 12.29 |
| DFS | 2.83 | 0.50 | 2.64 | 1.52 | 3.03 | 2.44 | 8.56 | 1.12 | 4.66 | 3.76 | 1.17 | 7.52 | 2.35 | 12.99 | 5.07 |
| SF | 0.56 | 1.61 | 0.36 | 0.06 | 1.65 | 1.12 | 0.06 | 6.51 | 1.50 | 2.72 | 4.19 | 3.50 | 0.45 | 1.28 | 0.79 |
| STT | 1.34 | 2.56 | 1.58 | 0.21 | 2.00 | 1.08 | 7.18 | 3.99 | 2.21 | 0.10 | 0.35 | 5.49 | 8.64 | 1.19 | 0.54 |
| CST | 2.37 | 0.74 | 0.65 | 0.98 | 0.93 | 2.46 | 3.97 | 7.25 | 2.42 | 1.77 | 0.31 | 0.68 | 3.56 | 0.30 | 2.96 |
| CSB | 2.27 | 0.34 | 1.03 | 1.67 | 2.05 | 0.89 | 4.49 | 9.20 | 3.77 | 0.13 | 2.13 | 0.16 | 0.21 | 0.95 | 5.01 |
| Eigen value | 6.62 | 5.34 | 5.13 | 4.18 | 3.49 | 3.25 | 2.95 | 2.91 | 2.55 | 2.52 | 2.40 | 2.31 | 2.23 | 2.12 | 2.03 |
| % variance | 7.35 | 5.93 | 5.69 | 4.64 | 3.88 | 3.61 | 3.28 | 3.24 | 2.83 | 2.79 | 2.67 | 2.57 | 2.48 | 2.35 | 2.26 |
| Cumulative | 7.35 | 13.28 | 18.97 | 23.62 | 27.50 | 31.10 | 34.38 | 37.62 | 40.45 | 43.24 | 45.91 | 48.48 | 50.95 | 53.30 | 55.56 |
| PC refers to the principal components. Leaf SPAD index at 21 days after transplanting (S21) and at 28 days (S28); length of main vine at 14 days after transplanting (LMV14) and at 21 (LMV21); accumulated degree-days for flowering (DDF); number of fruits per plant (NFP); average mass of fruits (MF); productivity of fruits (PF); height of fruit (HF); diameter of fruit (DF); thickness of fruit peel (TFP); resistance of fruit peel to penetration (RFP); resistance of fruit pulp to penetration (RP); thickness of fruit pulp (PT); diameter of internal cavity of fruit (DIC); total content of fruit pulp carotenoids (TC), and lutein (L); number of seeds per plant (NSF); mass of seeds per fruit (MSF); ratio of seed to fruit mass (MS/F); mass of one hundred seeds (MOH); productivity of seeds (PS); seed thickness (ST); seed length (SL); seed width (SW); seed oil content (SOC); seed oil productivity (SOP). Growth habit (GH); stem colour (SC); intensity of leaf green (ILG), leaf silvering (LS), intensity of leaf silvering (ILS); leaf serration (LS); presence of trichomes in the leaves (PAL); amount of trichomes in the adaxial surface of leaves (ATT); amount of trichomes in the abaxial surface of leaves (ATL); leaf recess (LR); presence of trichomes in the petiole (PTP); amount of trichomes in the petiole (ATP); green intensity of male pedicel (GIMP); format of fruits (FF); format of peduncle (FP); number of colours of fruit peel (NPC); topography of fruit surface (FT); format of floral scar (FFS); peel texture (PT); predominant colour of fruit peel (PC); depth of fruits slices (DFS); seed format (SF); aspect of seed tegument (AST); seed tegument texture (STT); colour of seed tegument (CST); colour of seed border (CSB). | | | | | | | | | | | | | | | |
